# Myocardial T1 mapping in different cardiomyopathies at 3.0T

**DOI:** 10.1186/1532-429X-14-S1-M13

**Published:** 2012-02-01

**Authors:** Juliano L Fernandes, Andreas Greiser, Strecker Ralph, Jose Alvaro Silva, Gabriel S Figueiredo, Jose Michel Kalaf, Otavio R Coelho-Filho

**Affiliations:** 1 University of Campinas, Campinas, Brazil; 2 Radiologia Clinica de Campinas, Campinas, Brazil; 3 MR Cardiology, Siemens Healthcare, Erlangen, Germany; 4 Healthcare MR, Siemens Ltda, Sao Paulo, Brazil

## Summary

Measure T1 times in patients with idiopathic dilated cardiomyopathy (DCM), hypertrophic cardiomyopathy (HCM), ischemic cardiomyopathy (ICM) and compare these values to normal controls using 3.0T CMR.

## Background

Diffuse myocardial fibrosis has been described in different cardiomyopathies and has recently been correlated to T1 times measured by cardiac magnetic resonance (CMR). Despite these advances it is yet unknown whether there are differences in T1 maps among these cardiomyopathies and if CMR can distinguish different levels of fibrosis in each category. Moreover, most work on T1 mapping has been carried on 1.5T scanners with limited data on these values at 3.0T.

## Methods

T1 maps were measured pre and 10 minutes after the infusion of 0.2mmol/kg of gadolinium on 80 subjects (20 normal controls, 20 patients with ICM, 20 patients with DCM and 20 patients with HCM). Images were obtained in a mid-ventricular short axis plane using a Modified Look Locker Inversion Recovery (MOLLI) sequence. All images with different inversion times were transferred to an off-line workstation and analyzed by drawing a region of interest around the entire myocardium and fitting the data after heart rate correction. Infarct areas in patients with ICM were excluded. Values obtained pre and post contrast were compared using General Linear Model with post-hoc LSD.

## Results

**Figure 1:**
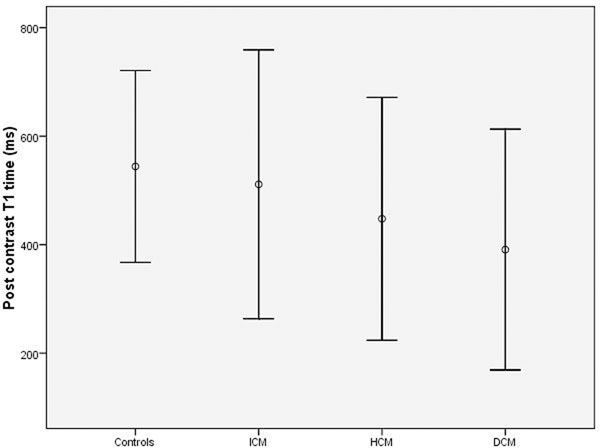
Mean and 2SD T1 times for controls and each type of cardiomyopathy.

The mean age of all subjects was 51.2±14.9 years with 66% men. After contrast, a significant difference was observed among the categories regarding T1 times (controls 544±88ms, ICM 511±124ms, HCM 448±112ms and DCM 390±111ms, P=0.004) - Figure 1. Patients with DCM had significantly lower T1 values compared to both controls and ICM (P=0.001 and P=0.02 respectively). HCM patients also differed significantly from controls (P=0.01) while patients with ICM did not differ from normal individuals (P=0.43). No significant differences were found among each category of cardiomyopathy or controls in T1 values pre contrast (controls 1243±71ms, ICM 1281±99ms, HCM 1209±88ms and DCM 1241±58ms, P=0.19).

## Conclusions

Our results show that post contrast T1 mapping can distinguish different type of cardiomyopathies from controls at 3.0T. DCM and HCM show greatest reductions in T1 times suggesting increased diffuse fibrosis as compared to ICM.

## Funding

Fundacao de Amparo a Pesquisa do Estado de Sao Paulo (FAPESP).

